# Chinese Clinical Named Entity Recognition From Electronic Medical Records Based on Multisemantic Features by Using Robustly Optimized Bidirectional Encoder Representation From Transformers Pretraining Approach Whole Word Masking and Convolutional Neural Networks: Model Development and Validation

**DOI:** 10.2196/44597

**Published:** 2023-05-10

**Authors:** Weijie Wang, Xiaoying Li, Huiling Ren, Dongping Gao, An Fang

**Affiliations:** 1 Institute of Medical Information and Library Chinese Academy of Medical Sciences & Peking Union Medical College Beijing China

**Keywords:** Chinese clinical named entity recognition, multisemantic features, image feature, Robustly Optimized Bidirectional Encoder Representation from Transformers Pretraining Approach Whole Word Masking, RoBERTa-wwm, convolutional neural network, CNN

## Abstract

**Background:**

Clinical electronic medical records (EMRs) contain important information on patients’ anatomy, symptoms, examinations, diagnoses, and medications. Large-scale mining of rich medical information from EMRs will provide notable reference value for medical research. With the complexity of Chinese grammar and blurred boundaries of Chinese words, Chinese clinical named entity recognition (CNER) remains a notable challenge. Follow-up tasks such as medical entity structuring, medical entity standardization, medical entity relationship extraction, and medical knowledge graph construction largely depend on medical named entity recognition effects. A promising CNER result would provide reliable support for building domain knowledge graphs, knowledge bases, and knowledge retrieval systems. Furthermore, it would provide research ideas for scientists and medical decision-making references for doctors and even guide patients on disease and health management. Therefore, obtaining excellent CNER results is essential.

**Objective:**

We aimed to propose a Chinese CNER method to learn semantics-enriched representations for comprehensively enhancing machines to understand deep semantic information of EMRs by using multisemantic features, which makes medical information more readable and understandable.

**Methods:**

First, we used Robustly Optimized Bidirectional Encoder Representation from Transformers Pretraining Approach Whole Word Masking (RoBERTa-wwm) with dynamic fusion and Chinese character features, including 5-stroke code, Zheng code, phonological code, and stroke code, extracted by 1-dimensional convolutional neural networks (CNNs) to obtain fine-grained semantic features of Chinese characters. Subsequently, we converted Chinese characters into square images to obtain Chinese character image features from another modality by using a 2-dimensional CNN. Finally, we input multisemantic features into Bidirectional Long Short-Term Memory with Conditional Random Fields to achieve Chinese CNER. The effectiveness of our model was compared with that of the baseline and existing research models, and the features involved in the model were ablated and analyzed to verify the model’s effectiveness.

**Results:**

We collected 1379 Yidu-S4K EMRs containing 23,655 entities in 6 categories and 2007 self-annotated EMRs containing 118,643 entities in 7 categories. The experiments showed that our model outperformed the comparison experiments, with *F*_1_-scores of 89.28% and 84.61% on the Yidu-S4K and self-annotated data sets, respectively. The results of the ablation analysis demonstrated that each feature and method we used could improve the entity recognition ability.

**Conclusions:**

Our proposed CNER method would mine the richer deep semantic information in EMRs by multisemantic embedding using RoBERTa-wwm and CNNs, enhancing the semantic recognition of characters at different granularity levels and improving the generalization capability of the method by achieving information complementarity among different semantic features, thus making the machine semantically understand EMRs and improving the CNER task accuracy.

## Introduction

### Background

Abundant medical data have been accumulated since the development of the hospital information system, among which the electronic medical records (EMRs) contain information closely related to patients’ diagnosis and treatment processes [1]. As important records of patients’ medical activities, effective extraction and use of the medical information contained in EMRs could provide clinical decision-making support for doctors and realize personalized medical guidance and health management for patients. It could also help biomedical researchers discover the tacit medical knowledge, thus providing ideas for studies of the association between diseases, the relationship between symptoms, the prediction of diseases and therapies, complication prediction, comorbidity analysis, etc. The medical information would be rapidly extracted from the unstructured EMRs through named entity recognition (NER). NER is a basic task of natural language processing, which will lay the foundation for the construction of medical knowledge graphs, medical knowledge bases, and so on by steps such as medical entity structuring, medical entity standardization, and medical entity relationship extraction. It will also provide fundamental support for practical application scenarios such as medical knowledge retrieval systems, clinical decision support systems, clinical event extraction, and so on [2,3].

Clinical NER (CNER) refers to the recognition of entities such as anatomy, disease, symptoms, clinical examination, medication, surgical procedure, and so on from EMRs [4,5]. Chinese CNER is more difficult than English NER for several reasons. First, Chinese words lack space segmentation and have blurred boundaries. Second, the composition of a Chinese entity is complex and may contain various figures, letters, and abbreviations. Third, Chinese grammar is complicated, and the same word may represent different entity types in different contexts. Therefore, Chinese CNER remains a research focus.

Recently, the features of radicals for Chinese characters have been widely used to improve the efficiency of different Chinese natural language processing tasks [6-8]. Chinese characters, known for thousands of years, are highly developed morpheme scripts that are still used worldwide with unique ideology [9]. Chinese characters include single-component and multiple-component characters. A single-component character cannot be divided, for example, “心 (heart),” “手 (hand),” and “口 (mouth),” and so on; whereas a multiple-component character is composed of basic components, accounting for >90% of Chinese characters [10], for example, the radical for “呕 (vomit)” and “吐 (vomit)” is “口 (mouth),” and the radical for “肿 (swelling)” and “胀 (swelling)” is “月 (month),” which refers to meat or organs in ancient times. Chinese characters are divided into associative compound characters, indicative characters, pictographic characters, and picto-phonetic characters based on their characteristics. In addition, Chinese characters are also called square characters, as they are square, and there are 8 structures of Chinese characters that are subdivided based on their intrinsic shape and construction. Therefore, Chinese characters contain rich deep semantic information. Applying radicals, phonological codes, shape structures, and other features would help to improve Chinese CNER accuracy.

The contributions of this study are as follows: (1) using pretrained language model (PLM) Robustly Optimized Bidirectional Encoder Representation from Transformers Pretraining Approach Whole Word Masking (RoBERTa-wwm) with a dynamic fusion transformer layer to obtain the semantic features of Chinese characters; (2) using CNNs for extracting the radicals and picto-phonetic features of Chinese characters through the 5-stroke code, Zheng code, phonological code, and stroke code; (3) converting Chinese characters into square images, extracting Chinese character image features from another modality by CNNs, and deeply capturing the pictographic characteristics of Chinese characters; and (4) improving the semantic recognition ability of the model at different levels of granularity, achieving information complementarity between different semantic features, and improving the effect and generalization ability of the model based on multisemantic features.

### Related Works

#### Medical NER

In recent decades, the medical NER is still a research focus. Medical NER research has 3 main development stages as follows: based on dictionaries and rules, based on statistical machine learning, and based on deep learning.

The dictionary-based [11-13] methods need to construct a domain dictionary in advance to achieve medical NER by matching algorithms. The accuracy of this method is relatively higher. However, it may be affected by the large number, strong specialization, and high complexity of Chinese medical entities. In addition, medical terminologies are updated quickly with the rapid development of the medical field, and the lack of new terminologies will also affect medical NER accuracy. The rule-based [14,15] methods need experts in a particular field to formulate the rule templates based on information such as context grammar and structure. However, the rules are poorly universal in different fields. The methods based on dictionaries and rules [16-18] are poorly generalized, time-consuming, and objective, as much time and labor are required. Therefore, many scholars have gradually applied methods based on statistical machine learning on medical NER. The commonly used methods include maximum entropy [19], support vector machine [20,21], hidden Markov model [22,23], and conditional random fields (CRF) [24,25]. However, these methods rely on large-scale annotation data sets [26] and manual feature selection [13,27]. Moreover, the quality of the selected features will directly affect the medical NER results.

With the continuous development of deep learning, Cocos et al [28] found that deep learning has advantages over traditional machine learning. It can automatically extract the characteristics of various levels and reduce the subjectivity of artificial feature selection. This thereby improves the result accuracy. The commonly used deep learning models include convolutional neural networks (CNNs) [29], recurrent neural networks [30], long short-term memory (LSTM) [31], Word to Vector (Word2Vec) [32], Bidirectional Encoder Representation from Transformers (BERT) [33], and so on. However, fully extracting the data features by using a neural network alone is challenging. Most scholars took Long LSTM-CRF as the main framework to make up for the medical NER deficiency using a single neural network [34]. The Bidirectional LSTM-CRF (BiLSTM-CRF) [35] model was then developed. This model could better capture contextual information as an important milestone in medical NER and has been widely used in the medical field [36,37]. To improve the ability to capture details and extract features of medical NER models, many studies added Word2Vec with static representation [38], Global Vectors for Word Representation [39] with static representation, Embeddings from Language Models (ELMo) [40,41] with dynamic representation, CNN [42], and attention mechanism [43] to the BiLSTM-CRF model. Some studies [44,45] have shown that the application of the BiLSTM-CRF model combined with the word vector generated by BERT could significantly improve medical NER accuracy. BERT provided a more accurate word representation and achieved better task results than traditional word vector methods. As per the specialty of medicine and the characteristics of Chinese characters involved, the clinical dictionaries, root-level features, parts of speech, radicals, and phonological codes have been added in the BiLSTM-CRF model in some studies [46-51] for improving Chinese CNER performance.

#### PLMs Technique

PLMs are pretrained on a large-scale corpus to obtain prior semantic knowledge from unlabeled text and improve the effectiveness of different downstream tasks. The word vector generated by a bidirectional language model BERT with stacked transformer substructures contains not only the preliminary information from the corpus training but also the encoded contextual information. Some robust versions of BERT have been constructed since BERT was proposed in 2018. For example, the RoBERTa model [52], which replaces the static (MASK) strategy with a dynamic (MASK) strategy, and the words (MASK) in each sequence dynamically change in different epoch trainings. In addition, the RoBERTa model is retrained with bigger batches and longer sequences, and the next-sentence prediction task, which is not related to the downstream task, is canceled during the pretraining. Compared with the BERT model, the RoBERTa model performs better on multiple natural language processing tasks. However, the character-level RoBERTa model does not fit the Chinese natural language processing, as the different segmentation modes between Chinese and English words suffer a limitation of lacking word information. Then, the word-level RoBERTa-wwm model [53] was proposed based on Chinese characteristics, which greatly improved the text representation ability in Chinese [54].

## Methods

### Data Collection

The Yidu-S4K data set, shared publicly by YiduCloud, is derived from the Chinese EMRs entity recognition task of the China Conference on Knowledge Graph and Semantic Computing 2019 [55]. It contains 1379 EMRs with 6 entity types, including Disease (medically defined disease and diagnoses made by physicians based on etiology, pathophysiology, pathological classification, and clinical staging); Anatomy (anatomical parts of the body where disease, signs, and symptoms occurred); Laboratory (physical or chemical tests performed by the laboratory department in clinical work); Image (imaging [x-ray, computed tomography, magnetic resonance imaging, positron emission tomography-computed tomography, etc], ultrasound, and electrocardiogram); Medicine (specific chemical substances used for disease treatment); and Operation (treatments focused on surgery such as excision and suturing performed by the physician locally on the patient’s body).

Self-annotated EMR data, collected from publicly desensitized Chinese EMR websites [56], contain 2007 EMRs. As per the Terminology of Clinical Medicine issued by the National Health Commission of the People’s Republic of China, we used the BIO (B signifies the beginning of an entity, I signifies that the word is inside an entity, and O signifies that the word is just a regular word outside of an entity) tagging method to pretag 7 entity types in the EMRs, including Disease (same definition as the Yidu-S4K data set); Symptoms (abnormal manifestations as perceived by the sensory organs of patients and physicians); Anatomy (same definition as the Yidu-S4K data set); Examination (includes imaging examinations and laboratory tests mentioned in the Yidu-S4K data set); Instrument (apparatus and mechanical equipment for disease prevention, diagnosis, treatment, health care, and rehabilitation); Medicine (the same definition as the Yidu-S4K data set); and Operation (same definition as the Yidu-S4K data set). Subsequently, 4 medical experts manually checked and corrected the tags. The interclass correlation efficient consistency test revealed that we had good annotation quality.

The ratio of the training set to the test set of the EMRs was 7:3. The Yidu-S4K data set was preprovided with 1000 EMRs as the training data sets (1000/1379, 72.52%) and 379 EMRs as the test data sets (379/1379, 27.48%). The self-annotated data set was divided by randomization into 1401 EMRs as the training data sets (1401/2007, 69.81%) and 379 EMRs as the test data sets (606/2007, 30.19%). Table 1 lists the details of the different types of entities in the 2 EMR data sets.

**Table 1:** The statistics of different types of entities in 2 electronic medical record data sets

Data sets and entity type			Training set, n		Test set, n
**Yidu-S4K**					
	Disease	4212		1323	
	Anatomy	8426		3094	
	Laboratory	1195		590	
	Image	969		348	
	Medicine	1822		485	
	Operation	1029		162	
	All entities	17,653		6002	
**Self-annotated**					
	Disease	9470		4504	
	Symptoms	26,334		11,065	
	Anatomy	17,877		7588	
	Examination	19,664		8746	
	Instrument	1244		560	
	Medicine	5314		2566	
	Operation	2578		1133	
	All entities	82,481		36,162	

### Ethical Considerations

Ethics approval was not required because the patient’s private information was masked by the website.

### Experiments Settings

In this study, all the experiments were conducted by Python [57] and PyTorch [58]. Table 2 shows the experimental parameters. The experiments used *RoBERTa-wwm-ext-large* model pretraining data, optimized parameters using Adam W, dropout to prevent overfitting, the batch size of 32, BiLSTM hidden layer dimension of 768, maximum sequence length of 510, RoBERTa-wwm dimension of 768, semantic feature dimension of 124, and image feature dimension of 128. On 2 Chinese CNER data sets, we used the same parameters.

**Table 2:** Parameter settings.

Parameter	Value
Dropout	0.5
Epoch	Optimization
Optimization	Adam W
Learning rate	0.0001
Batch size	32
BiLSTM^a^ hidden layer	768
Max_len	510
RoBERTa-wwm^b^ feature dimension	768
Semantic feature dimension	124
Image feature dimension	128

^a^BiLSTM: Bidirectional Long Short-Term Memory.

^b^RoBERTa-wwm: Robustly Optimized Bidirectional Encoder Representation from Transformers Pretraining Approach Whole Word Masking.

### Evaluation Metrics

The experiments used precision, recall, and *F*_1_-score to evaluate the model performance. The formulas for each index are as follows:


*Precision = TP / (TP + FP)*
**(1)**

*Recall = TP / (TP + FN)*
**(2)**

*F_1_-score = (2 × precision × recall) / (precision + recall)*
**(3)**

where precision is the proportion of positive samples in all samples predicted to be positive; recall is the proportion of positive samples in all positive samples; *F*_1_-score is the harmonic mean of precision and recall; true positive (TP) is the number of positive samples predicted to be positive, that is, the number of correctly recognized entities; false positive (FP) is the number of negative samples predicted to be negative, that is, the number of incorrectly recognized other texts as entities; and false negative (FN) is the number of positive samples predicted to be negative, that is, the number of unrecognized entities.

### Model Overview

**Figure 1:**
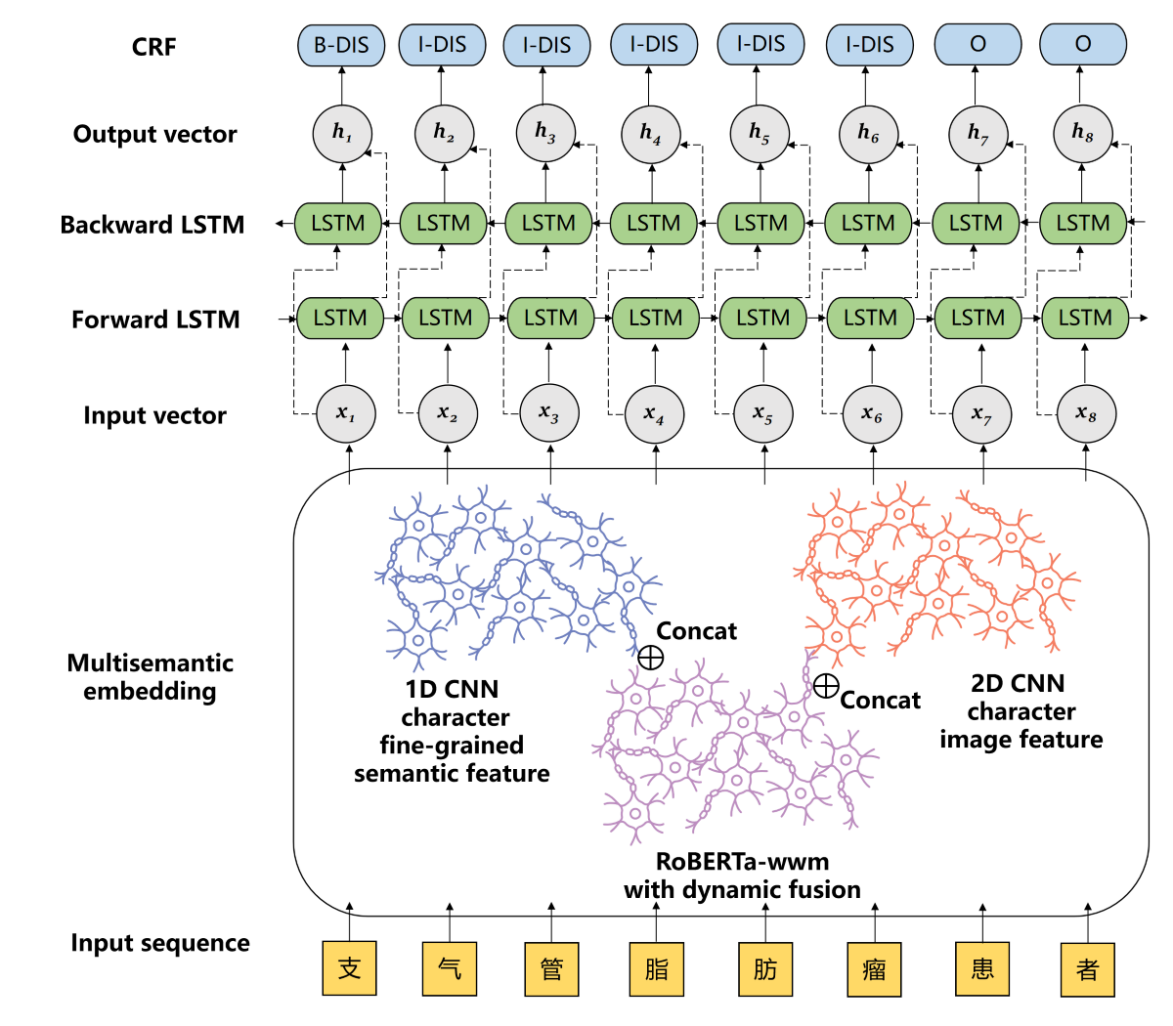
The main architecture of our model. 1D CNN: 1D convolutional neural network; 2D CNN: 2D convolutional neural network; B-DIS: beginning of disease entity; CRF: conditional random fields; h: embedding of output character; I-DIS: inside of disease entity; LSTM: long short-term memory; O: other type; RoBERTa-wwm: Robustly Optimized Bidirectional Encoder Representation from Transformers Pretraining Approach Whole Word Masking; x: embedding of input character.

In this study, we proposed a CNER model based on multisemantic features, as shown in Figure 1. First, we used RoBERTa-wwm, the PLM, to obtain the embedded representation at the word level. Dynamic fusion is performed on the semantic representation generated by each transformer layer to make full use of RoBERTa-wwm representation information. Then, the embedded Chinese character fine-grained feature representation, including the 5-stroke code, Zheng code, phonological code, and stroke code, is extracted by 1D CNN, whereas the embedded Chinese character image representation is extracted from another modality by 2D CNN, with the Chinese characters as square images. Finally, the above multisemantic vectors were input into the BiLSTM layer for encoding and were decoded in the CRF layer to predict the tag probability.

### Multisemantic Embedding Layer

#### Overview

Many Chinese characters have retained their original connotations, as they originated from pictographic characters in ancient times. Moreover, the inherent fine-grained character information contained in Chinese characters often implies more additional semantic information. Accordingly, we obtained the 5-stroke code, Zheng code, phonological code, and stroke code information, as shown in Table 3, of the Chinese characters from ZDIC [59] and embedded them in the model. In addition, Chinese characters are squares, and different shapes and structures express different types of information. Characters with similar intrinsic characteristics may have similar meanings. Therefore, we took Chinese characters as graphics and obtained semantic information on Chinese character connotations from another modality. Multisemantics could obtain information comprehensively and learn a better feature representation by making use of information complementarity and eliminating the redundancy among different semantic features compared with a single-semantic feature, resulting in a more generalized model.

**Figure 2:**
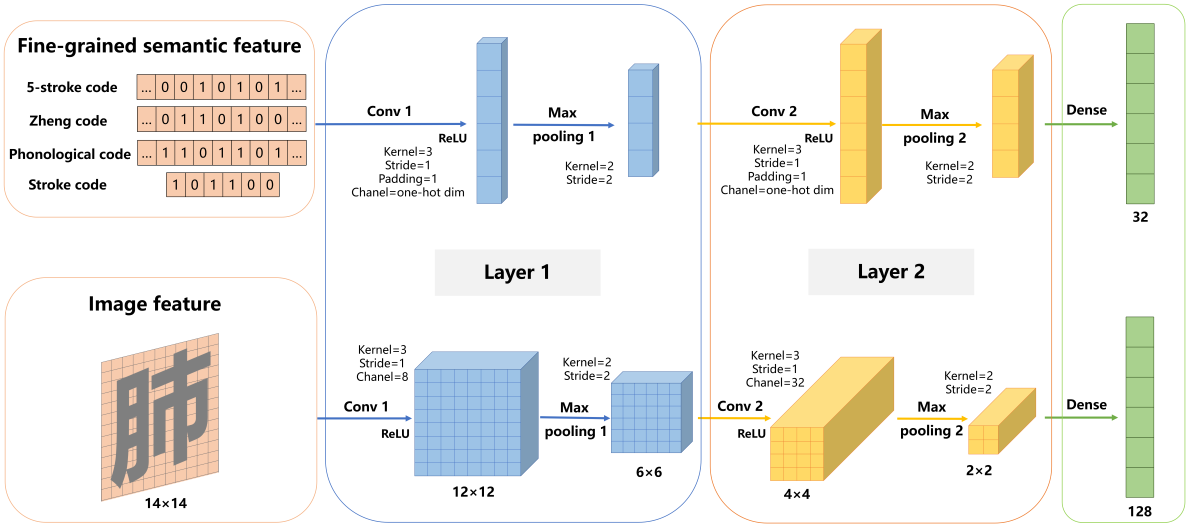
The process of obtaining Chinese character multisemantic features by convolutional neural network. ReLU: Rectified Linear Unit function; Conv 1: first convolutional layer; Conv 2: second convolutional layer; Max pooling 1: first max pooling layer; Max pooling 2: second max pooling layer; Dense: dense layer.

As shown in Figure 2, we converted the Chinese character 5-stroke code, Zheng code, phonological code, and stroke code into one-hot vector encoding and interpreted the Chinese characters as 14×14 images. Subsequently, we used a 2-layer CNN deeply extracting the Chinese character multisemantic features. Through the Convolution layer with the *ReLU* activation function, max pooling layer, and dense layer, we obtained the multisemantic vectors that could be embedded in the BiLSTM layer.

**Table 3:** Example of Chinese characters’ coded information from ZDIC.

Character	5-stroke code	Zheng code	Phonological code	Stroke code
呕 (vomit)	kaqy	jhos	ǒu	2,511,345
吐 (vomit)	kfg	jbvv	tù	251,121
肿 (swelling)	ekhh	qji	zhǒng	35,112,512
胀 (swelling)	etay	qch	zhàng	35,113,154
心 (heart)	nyny	wz	xīn	4544
手 (hand)	rtgh	md	shǒu	3112

#### RoBERTa-wwm With Dynamic Fusion

**Figure 3:**
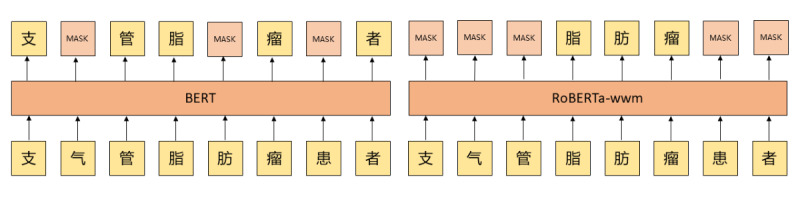
Mask process of Bidirectional Encoder Representation from Transformers (BERT) and Robustly Optimized Bidirectional Encoder Representation from Transformers Pretraining Approach Whole Word Masking (RoBERTa-wwm).

When RoBERTa-wwm pretrains the corpus, it is segmented on the language technology platform established by the Harbin Institute of Technology based on Wikipedia content in Chinese, which can provide a basis for achieving wwm. As shown in Figure 3, the word “支气管 (bronchi)” in the RoBERTa-wwm model is completely masked by random wwm, whereas only single characters can be randomly masked in the BERT model, for example, only 1 character “气 (gases)” was masked in the word “支气管 (bronchi).” Thus, the RoBERTa-wwm model can learn the word-level semantic representations in Chinese.

**Figure 4:**
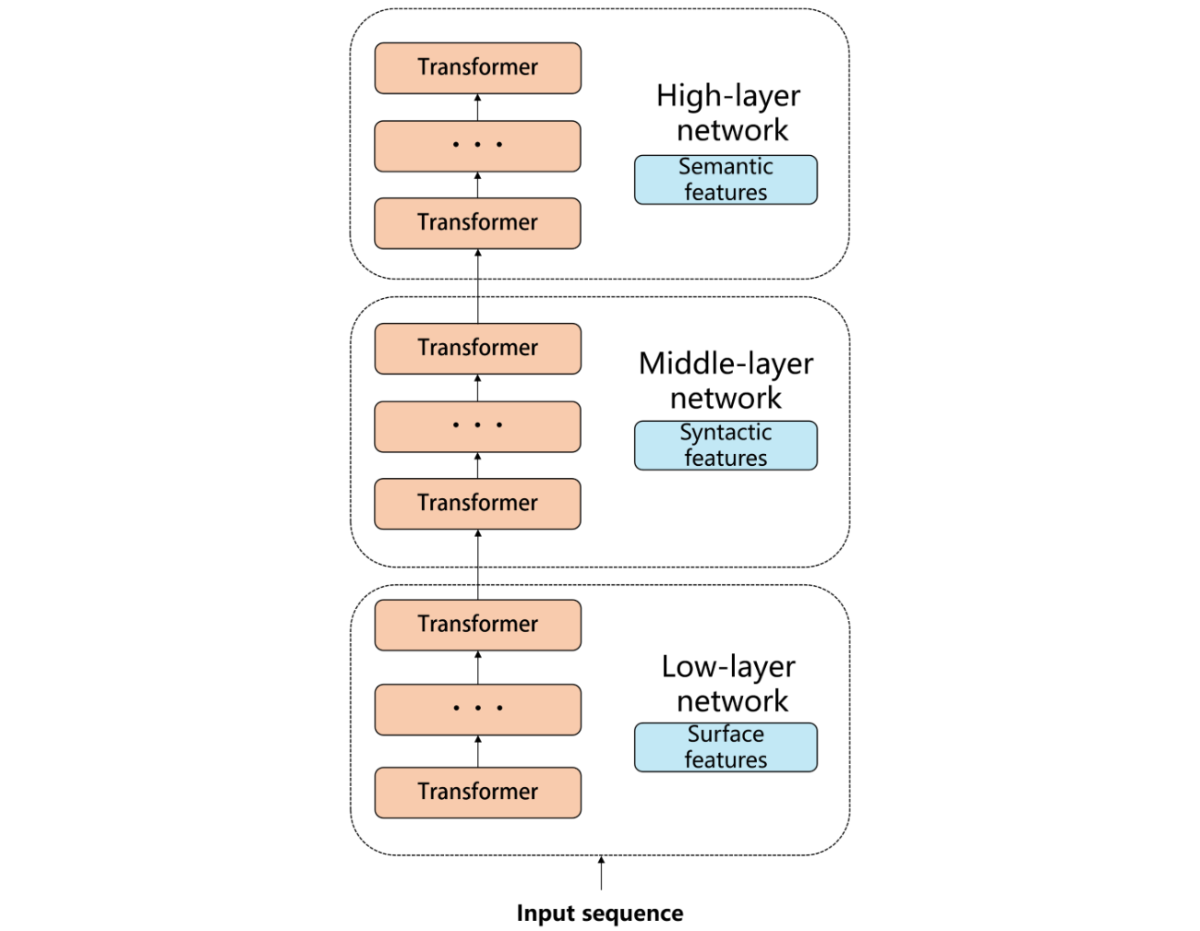
Coding representation of Transformer with 12 layers of Bidirectional Encoder Representation from Transformers model.

The encoder structure of each transformer layer of the BERT model outputs had different abstract representations of grammar, semantics, and real knowledge in sentences. Studies have confirmed that each layer of the BERT model represents text information differently through 12 natural language processing tasks [60]. As shown in Figure 4, the low transformer mainly learns and encodes surface features; the middle transformer learns and encodes syntactic features; and the high transformer learns and encodes semantic features.

The transformer structure of the RoBERTa-wwm model is consistent with that of the BERT model. To make full use of the representation information of each transformer layer, we used the RoBERTa-wwm model with dynamic fusion [61]. This helped in assigning the initial weight to the representation vector of 12 transformer layers, determining the weight during training, and weighing the representation vector generated by each layer.

Assume that the text input sequence *seq* = (*x*_1_,*x*_2_,*x*_3_, …, *x*_n_), where n is the total length of the character contained in the sequence; *x_i_* is the *i*^th^ character of the input sequence; and the fusion formula is as follows:

*v_i_^RoBERTa-wwm^=Dense_unit=512_ (x_i_)* {**∑**^n^_i=1_
*α_c_ × h_c_}, (c ∈ [1,12])* **(4)**

*v_i_^(RoBERTa-wwm)^* is the output representation by the RoBERTa-wwm model with dynamic fusion for the current character *x_i_*; *h_c_* is the output representation by each transformer layer of the RoBERTa-wwm model, and *α_c_* is the output weight value assigned to each layer by RoBERTa-wwm.

#### Fine-Grained Semantic Feature

##### 5-Stroke Code

The 5-stroke code is a typical semantic code, which encodes Chinese characters according to strokes and structures. Currently, it is widely used to code Chinese characters. The expression of the 5-stroke code may inevitably repeat with the phonological code, for example, the 5-stroke code for “亦 (also)” is “you,” while the phonological code for “亦 (also)” is also “you” [62]. Hence, we combined the 5-stroke code and Zheng code to compensate for the encoding deficiency. We used the 5-stroke code in Zdic.net to vectorize the Chinese characters using the following formulas:


*p = f_fc_ (seq)*
**(5)**

*vifc=efc (pi), (i∈Z ∩ i∈ [1,n])*
**(6)**

where *f_fc_* represents the function that maps the input character sequence into the 5-stroke code and *v_i_^fc^* represents the 5-stroke code vector corresponding to *x_i_*.

##### Zheng Code

The Zheng code was created by famous Chinese literature professors as per the strokes and roots of Chinese characters through in-depth research on the patterns and structures of Chinese characters. The early Microsoft operating system in Chinese adopted the Zheng code as the built-in code. This indicates that Zheng code is a scientific coding of Chinese characters. Chinese characters with similar codes may contain related semantic information. Hence, the potential semantic relationship of text may be found by mining the structural information of Chinese characters using Zheng code. The Zheng code was vectorized as the 5-stroke code and has the following formulas:


*p = f_zc_ (seq)*
**(7)**

*vizc=ezc (pi), (i∈Z ∩ i∈ [1,n])*
**(8)**

where *f_zc_* represents the function that maps the input character sequence into the Zheng code and *v_i_^zc^* represents the Zheng code vector corresponding to *x_i_*.

##### Phonological Code

Over 90% of Chinese characters are picto-phonetic characters [63]. Hence, pronunciation plays an important role in the semantic expressions of Chinese characters. We used the Pypinyin toolkit to vectorize the phonological code of Chinese characters using the following formulas:


*p = f_pc_ (seq)*
**(9)**

*vipc=epc (pi), (i∈Z ∩ i∈ [1,n])*
**(10)**

where *f_pc_* represents the function that maps the input character sequence into the phonological code and *v_i_^pc^* represents the phonological vector corresponding to *x_i_*.

##### Stroke Code

Chinese characters with similar strokes may have similar meanings. The strokes of each Chinese character were encoded in ZDIC [59], where 1, 2, 3, 4, and 5 represent the horizontal stroke, vertical stroke, left-falling stroke, right-falling stroke, and turning stroke, respectively. We transformed the stroke code into a 5-dimension vector, where each dimension was the corresponding number of strokes. The stroke code was vectorized in the same manner as the 5-stroke code and has the following formulas:


*p = f_sc_ (seq)*
**(11)**

*visc=esc (pi), (i∈Z ∩ i∈ [1,n])*
**(12)**

where *f_sc_* represents the function that maps the input character sequence into the stroke code and *v_i_^sc^* represents the stroke code vector corresponding to *x_i_*.

To extract the fine-grained semantic features of Chinese characters deeply, we trained the character features using CNNs. The character features were trained by 2 convolutions with a kernel of 3 and *ReLU* function as well as max pooling of 2×2, where the number of output channels was the dimension of each feature vector. Finally, the 32-dimension Chinese character vector was obtained through a full connection in the dense layer, as shown in Figure 2.

#### Image Feature

Chinese characters have been derived from pictographic symbols since ancient times, and characters with similar symbolic appearances have similar image features. However, the fonts of Chinese characters have changed a lot over time. Simplified characters have lost much pictographic information compared with complex characters. Therefore, Cui et al [64] used Chinese character images to extract Chinese character features and achieved better performance. Wu et al [65] tried different character fonts and found that the best result was obtained by using the *NotoSansCJKsc-Regular* font. On the basis of these findings, we used the Python Imaging Library to convert *NotoSansCJKsc-Regular* Chinese characters into black-and-white images and extracted image features by 2D CNN in depth as per the following formulas:


*e^if_1^=(Max pooling 1 (Conv 1 (K ⊗ H))*
**(13)**

*e^if_2^=(Max pooling 2 (Conv 2 (K ⊗ H)*
**(14)**

*v_i_^if^*=Dense (*e^if_2^*) **(15)**

where *K* is a kernel; *H* is the original embedded image matrix; *Conv 1*, *Max pooling 1*, *Conv 2*, and *Max pooling 2* are the first convolutions with a kernel of 3 and channel of 8, the first max pooling with the kernel of 2×2, the second convolution with the kernel of 3 and channel of 32, and the second max pooling with the kernel of 2×2, respectively; *e^if_1^* is the result after the first convolution; *e^if_2^* is the result after the second convolution; Dense is the process of realizing the full connection; and *v_i_^if^* is the final 128-dimension Chinese character image vector trained by convolution, as shown in Figure 2.

Finally, the multisemantic features *v_i_^RoBERTa-wwm^*, *v_i_^fc^*, *v_i_^zc^*, *v_i_^pc^*, *v_i_^sc^*, and *v_i_^if^* were embedded by the array Concat function. The formula used is as follows:

*v_i_^input^* =Concat (*v_i_^RoBERTa-wwm^*, *v_i_^fc^*, *v_i_^zc^*, *v_i_^pc^*, *v_i_^sc^*, *v_i_^if^*) **(16)**

### BiLSTM Layer

The role of BiLSTM [66] is essential in NER. As shown in Figure 1, the forward LSTM and backward LSTM are responsible for memorizing the previous and subsequent text information, respectively. By combining the 2, contextual information can be obtained simultaneously, which helps to capture the bidirectional semantic dependency information in the text. The formulas used are as follows:


*h_i_^forward^=LSTM^forward^(α^<i-1>^, x_i_)*
**(17)**

*h_i_^backward^=LSTM^backward^(α^<i-1>^, x_i_)*
**(18)**

*h_i_=*[*h_i_^forward^; h_i_^backward^*] **(19)**

where *α^<i>^* represents the hidden layer state of the current memory cell; *LSTM^forward^* is the feature representation from front to back; *LSTM^backward^* is the feature representation from back to front; *h_i_^forward^* is the forward semantic information obtained through the forward LSTM at the *i*-th character position; *h_i_^backward^* is the backward semantic information obtained through the backward LSTM at the *i*-th character position; and *h_i_* represents a combination of hidden states in both.

### CRF Layer

The BiLSTM can be used to handle contextual relationships. However, it cannot consider the dependencies between tags. Therefore, it is necessary to add a constraint relation for the final predicted label by using the CRF [67] layer to ensure the predicted label rationality. Given an input sequence where *X=*{*x_1_,x_2_,...,x_n_*}, we assume that the training output label sequence is *Y=*{*y_1_,y_2_,...,y_n_*}, where *n* is the number of model labels. The sequence score of the label and the probability of the label sequence *y* are calculated as follows:


*P (y|X)=e^∑i=1^ (Z_yi,yi+1_ + P_i+1,yi+1_) / (∑_(y ~∈Yx)_ e^∑i=1^ (Z_yi,yi+1_ + P_i+1,yi+1_)*
**(20)**

where *Z* is the transfer matrix; *Z_yi,yi+1_* is the score of the label transfer from *y_i_* to *y_i+1_*; *P_i+1,yi+1_* is the score of label *y_i+1_* corresponding to the *i+1*th character of the input sequence; *Y_x_* is the set of all possible label sequences. The final label of the output sequence is the set of labels with the highest probability.

Finally, we predicted the best label sequences by using the Viterbi algorithm [68] with the following formula:


y*=argmax(s(X, y))
**(21)**

## Results

To get convincing experimental results, we ran each model 5 times and calculated the average precision, average recall, and average *F*_1_-score.

### Performance Comparison With Ensemble Models

To verify the validity of the model, we compared our model with the existing ensemble models BiLSTM-CRF, ELMo-Lattice-LSTM-CRF, ELMo-BiLSTM-CRF, all CNNs, ELMo-encoder from transformer-CRF, and multigranularity semantic dictionary and multimodal tree-NER on Yidu-S4K and self-annotated data sets, and the results are shown in Table 4. The *F*_1_-scores of the experimental model on the Yidu-S4K data set were 18.31%, 4.15%, 4.26%, 4.12%, 3.69%, and 2.59% higher than those of the BiLSTM-CRF, all CNNs, ELMo-Lattice-LSTM-CRF, ELMo-BiLSTM-CRF, ELMo-encoder from transformer-CRF, and multigranularity semantic dictionary and multimodal tree-NER models, respectively. On the self-annotated data set, it was 5.14% higher than that of the BiLSTM-CRF. The results showed that the performance of the experimental model is superior to that of the existing model.

**Table 4:** Performance comparison of ensemble models on the Yidu-S4K and self-annotated data sets.

Data set and model			Precision (%)		Recall (%)		*F*_1_-score (%)
**Yidu-S4K**							
	BiLSTM-CRF^a^ [64]	69.43		72.58		70.97	
	ACNN^b^ [69]	83.07		87.29		85.13	
	ELMo^c^-lattice-LSTM-CRF [70]	84.69		85.35		85.02	
	ELMo-BiLSTM-CRF [41]	—^d^		—		85.16	
	ELMo-ET^e^-CRF [71]	82.08		86.12		85.59	
	MSD_DT_NER^f^ [72]	86.09		87.29		86.69	
	Our model	90.37		88.22		89.28	
**Self-annotated**							
	BiLSTM-CRF	81.98		77.10		79.47	
	Our model	84.24		84.99		84.61	

^a^BiLSTM-CRF: Bidirectional Long Short-Term Memory-conditional random fields.

^b^ACNN: all convolutional neural network.

^c^ELMo: Embeddings from Language Models.

^d^Not available.

^e^ET: encoder from transformer*.*

^f^MSD_DT_NER: multigranularity semantic dictionary and multimodal named entity recognition.

### Performance Comparison With PLMs Related to BERT

The performance of the PLM, BERT, is a milestone in natural language processing. To verify the BERT robust version’s validity of the RoBERTa-wwm model, we compared our model with the existing ensemble models with the BiLSTM-CRF, BERT-BiLSTM-CRF, and RoBERTa-wwm-BiLSTM-CRF on Yidu-S4K and self-annotated data sets, and the results are shown in Table 5. The *F*_1_-scores of the experimental model on the Yidu-S4K data set were 18.31%, 2.99%, and 0.82% higher than those of the BiLSTM-CRF, BERT-BiLSTM-CRF, and RoBERTa-wwm-BiLSTM-CRF models, respectively, and 5.14%, 2.95%, and 1.07% higher on the self-annotated data set, respectively.

**Table 5:** Performance comparison of PLMs^a^ on the Yidu-S4K and self-annotated data sets.

Data set and model			Precision (%)		Recall (%)		*F*_1_-score (%)
**Yidu-S4K**							
	BiLSTM^b^-CRF^c^ [64]	69.43		72.58		70.97	
	BERT^d^-BiLSTM-CRF	89.07		83.67		86.29	
	RoBERTa-wwm^e^-BiLSTM-CRF	90.08		86.90		88.46	
	Our model	90.37		88.22		89.28	
**Self-annotated**							
	BiLSTM-CRF	81.98		77.10		79.47	
	BERT-BiLSTM-CRF	82.48		80.86		81.66	
	RoBERTa-wwm-BiLSTM-CRF	84.23		82.86		83.54	
	Our model	84.24		84.99		84.61	

^a^PLM: pretrained language model.

^b^BiLSTM: Bidirectional Long Short-Term Memory.

^c^CRF: conditional random fields.

^d^BERT: Bidirectional Encoder Representation from Transformers.

^e^RoBERTa-wwm: Robustly Optimized Bidirectional Encoder Representation from Transformers Pretraining Approach Whole Word Masking.

### Performance Comparison of Each Entity

To comprehensively evaluate our model, we calculated the *F*_1_-score for each entity type on the Yidu-S4K and self-annotated data sets, as shown in Tables 6 and 7. The *F*_1_-score of our model on the Yidu-S4K data set for each of the 6 entity categories, except for the Image entity, increased by 0.2% to 7.6% compared with the data listed in the tables. The *F*_1_-score for the Image entity was 0.35% lower than that of the ELMo-BiLSTM-CRF model. However, the *F*_1_-scores for the Laboratory entity and Operation entity were 7.6% and 7.54% higher than those of the ELMo-BiLSTM-CRF model, respectively. The overall *F*_1_-score was 4.12% higher than that of the ELMo-BiLSTM-CRF model. For the self-annotated data set, our model improved each entity in 7 categories ranging from 0.09% to 14.49% over the listed data, with a greater improvement for Instrument entities.

**Table 6:** Performance comparison of each entity category on the Yidu-S4K data set.

Model	*F*_1_-score for each category (%)						
	All	Disease	Anatomy	Image	Laboratory	Medicine	Operation
ELMo^a^-BiLSTM^b^-CRF^c^ [41]	85.16	82.81	85.99	88.01	75.65	94.49	86.79
BERT^d^-BiLSTM-CRF	86.29	87.14	86.36	83.43	77.98	89.46	93.11
BERT-wwm^e^-BiLSTM-CRF	87.12	86.18	85.47	81.52	79.69	90.14	92.49
RoBERTa^f^-wwm-BiLSTM-CRF	88.46	87.71	87.01	86.69	82.36	93.22	92.87
Our model	89.28	87.91	87.47	87.66	83.25	94.98	94.33

^a^ELMo: Embeddings from Language Models.

^b^BiLSTM: Bidirectional Long Short-Term Memory.

^c^CRF: conditional random fields.

^d^BERT: Bidirectional Encoder Representation from Transformers.

^e^wwm: Whole Word Masking.

^f^RoBERTa: Robustly Optimized Bidirectional Encoder Representation from Transformers Pretraining Approach.

**Table 7:** Performance comparison of each entity category on the self-annotated data set.

Model	*F*_1_-score for each category (%)							
	All	Disease	Symptoms	Anatomy	Examination	Instrument	Medicine	Operation
BERT^a^-BiLSTM^b^-CRF^c^	81.66	81.33	85.87	83.86	90.36	60.38	89.72	79.75
BERT-wwm^d^-BiLSTM-CRF	81.58	74.91	83.89	81.23	88.84	54.76	85.63	68.49
RoBERTa^e^-wwm-BiLSTM-CRF	83.54	81.99	86.69	84.68	91.21	66.01	91.04	81.17
Our model	84.61	82.34	86.93	85.62	91.30	69.25	91.28	82.49

^a^BERT: Bidirectional Encoder Representation from Transformers.

^b^BiLSTM: Bidirectional Long Short-Term Memory.

^c^CRF: conditional random fields.

^d^wwm: Whole Word Masking.

^e^RoBERTa: Robustly Optimized Bidirectional Encoder Representation from Transformers Pretraining Approach.

### Ablation Analysis

#### Ablation Experiments for Multisemantic Features

**Figure 5:**
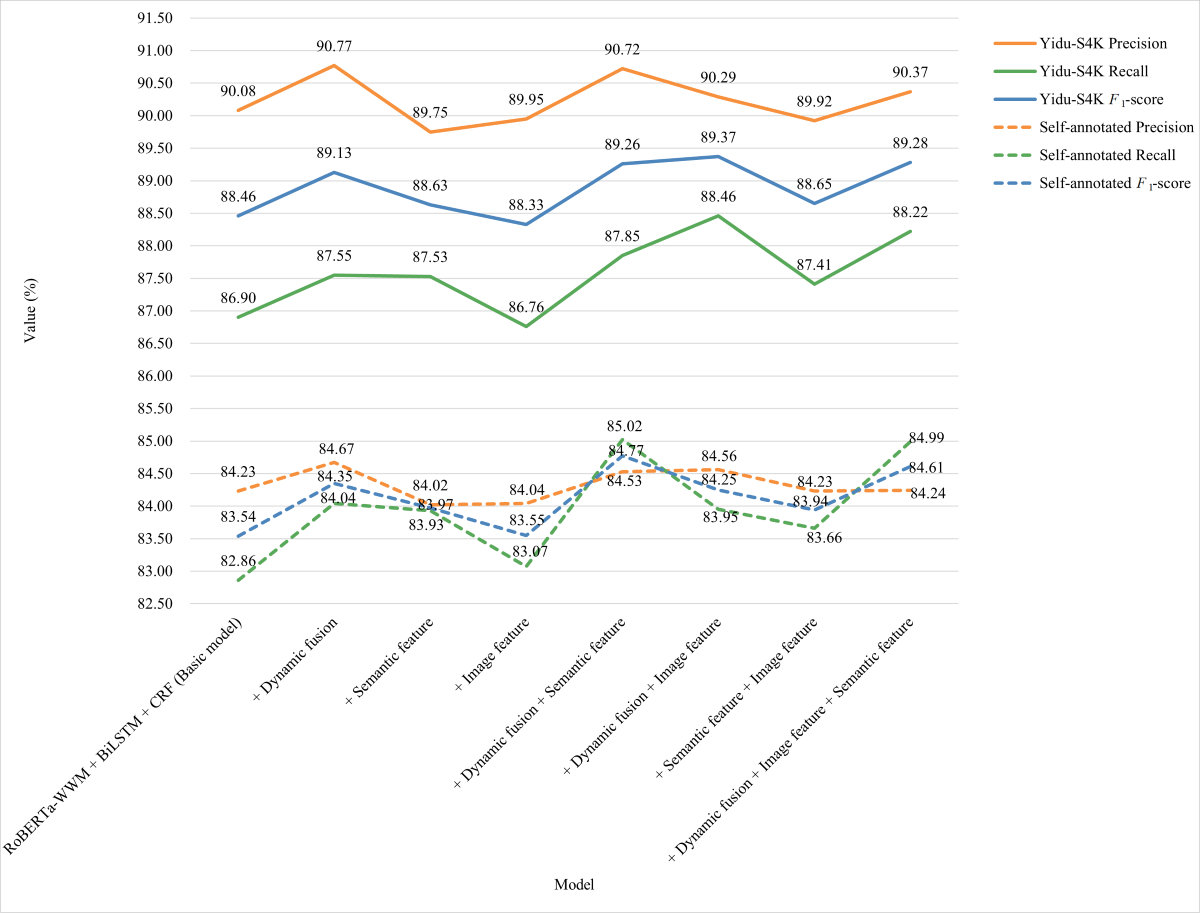
The results of ablation experiments for mutisemantic features on the Yidu-S4K and self-annotated data sets. BiLSTM: Bidirectional Long Short-Term Memory; CRF: Conditional Random Fields; RoBERTa-wwm: Robustly Optimized Bidirectional Encoder Representation from Transformers Pretraining Approach Whole Word Masking.

To verify the fine-grained semantic features and image features of Chinese characters, dynamic fusion was effective. We used the RoBERTa-wwm-BiLSTM-CRF model as the baseline to perform ablation experiments for the above contents on 2 EMR data sets, and the results are shown in Figure 5.

The performance of the model was significantly improved with the dynamic fusion of RoBERTa-wwm. After incorporating the semantic features of Chinese characters into the model alone, the overall performance of the model was not as high as that after dynamic fusion. However, the performance on both data sets was superior to that of the baseline. The performance of the model was unstable when image features of Chinese characters were added to the model alone. On the Yidu-S4K data set, the model’s performance was inferior to that of the baseline, whereas on the self-annotated data set, the model’s performance only improved slightly. After adding the semantic and image features of Chinese characters to the model, the performance of the model on the Yidu-S4K data set was superior to that of the baseline. Furthermore, it was better than that of the model with semantic or image features of Chinese characters alone. The performance of the model on the self-annotated data set was superior to that of the baseline and better than that of the model with the image features of Chinese characters alone. When the model combined dynamic fusion with the semantic features and image features of Chinese characters, it was found that the performance of the model was significantly improved on the 2 data sets. Dynamic fusion with image features of Chinese characters showed the best comprehensive performance on the Yidu-S4K data set, whereas dynamic fusion with semantic features of Chinese characters achieved the best comprehensive performance on the self-annotated data set. After combining the semantic and image features of the Chinese characters and dynamic fusion, it was noted that the performance of the model was superior to that of the baseline. Because the quality of the self-annotated EMRs is inferior to that of the public Chinese EMRs corpus and the self-annotated data set contains a wider coverage of departments, the comprehensive effect of the self-annotated data set is lower than that of the YiduS4K data set in Figure 5.

#### Ablation Experiments for Fine-Grained Semantic Features

**Figure 6:**
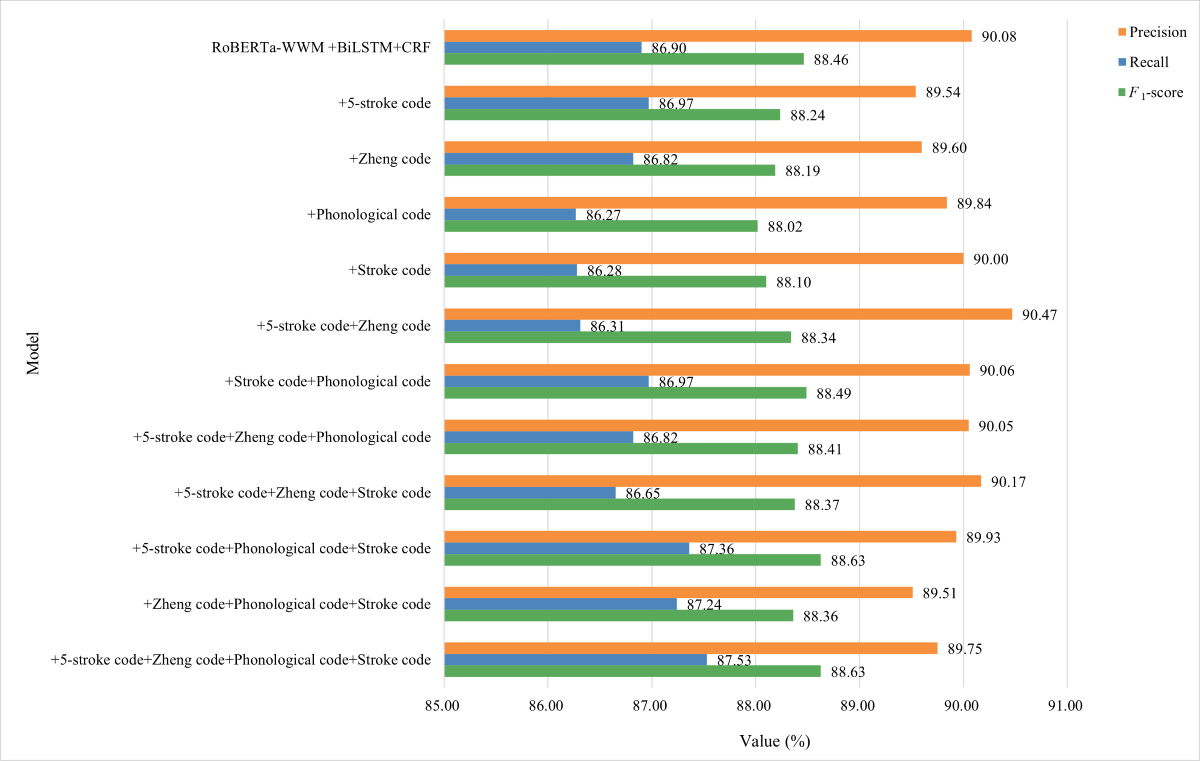
The results of ablation experiments for fine-grained semantic features on the Yidu-S4K data set. BiLSTM: Bidirectional Long Short-Term Memory; CRF: conditional random fields; RoBERTa-wwm: Robustly Optimized Bidirectional Encoder Representation from Transformers Pretraining Approach Whole Word Masking.

**Figure 7:**
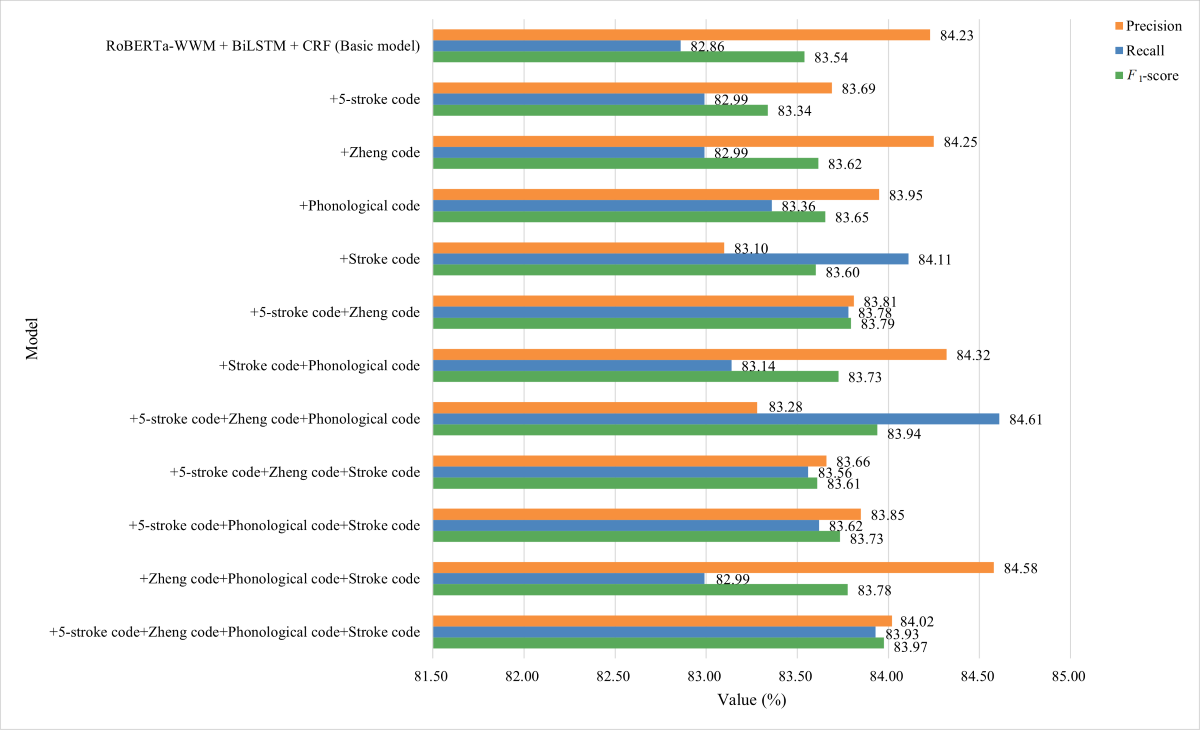
The results of ablation experiments for fine-grained semantic features on the self-annotated data set. BiLSTM: Bidirectional Long Short-Term Memory; CRF: conditional random fields; RoBERTa-wwm: Robustly Optimized Bidirectional Encoder Representation from Transformers Pretraining Approach Whole Word Masking.

The fine-grained semantic features of Chinese characters used in this study included the 5-stroke code, Zheng code, phonological code, and stroke code. To verify the effectiveness of these features, we used the RoBERTa-wwm-BiLSTM-CRF model as the baseline to perform ablation experiments for the 4 features on the 2 EMR data sets, and the results are shown in Figure 6 and Figure 7.

The *F*_1_-score of the model on the Yidu-S4K data set ranked in the top 2 for the 5-stroke code and Zheng code, whereas the *F*_1_-score on the self-annotated data set ranked in the top 2 for the phonological code or Zheng code. The performance of the model combining 2 features (the combination of 5-stroke code and Zheng code or the combination of phonological code and stroke code) was better than that of the model with only 1 feature, regardless of the data set. On the Yidu-S4K data set, the model combining 5-stroke code+phonological code+stroke code showed the best comprehensive performance, followed by the combinations of 5-stroke code+Zheng code+phonological code, 5-stroke code+Zheng code+stroke code, and Zheng code+phonological code+stroke code. On the self-annotated data set, the model combining the 5-stroke code+Zheng code+phonological code showed the best comprehensive performance, followed by Zheng code+phonological code+stroke code, 5-stroke code+phonological code+stroke code, and 5-stroke code+Zheng code+stroke code. On the Yidu-S4K data set, only the model combining the 5-stroke code+phonological code+stroke code showed a comprehensive performance superior to that of the baseline. However, on the self-annotated data set, the comprehensive performance of all combinations was superior to that of the baseline. The performance of the model combining 3 features was less stable. The model combined 4 features on the Yidu-S4K and self-annotated data sets and achieved the best comprehensive performance among all the combinations.

### Error Analysis

From Tables 6 to Table 7, our model improved the entity recognition performance of each entity category to different degrees. However, the entity recognition effect differs for each category. The *F*_1_-scores of Disease, Anatomy, Image, Laboratory, Medicine, and Operation entity recognition on the Yidu-S4K data set were 87.91%, 87.47%, 87.66%, 83.25%, 94.98%, and 94.33%, respectively. The *F*_1_-scores of Disease, Symptoms, Anatomy, Examination, Instrument, Medicine, and Operation entity recognition on the self-annotated data set were 82.34%, 86.93%, 85.62%, 91.31%, 69.25%, 91.28%, and 82.49%, respectively. On the Yidu-S4K data set, the precision of Laboratory entity recognition was the lowest, followed by the Anatomy entity, Image entity, and Disease entity. On the self-annotated data set, the precision of Instrument entity recognition was the lowest, followed by the Disease entity, Anatomy entity, and Operation entity. We concluded the following 7 main causes of the errors that occurred based on a review of the data set and model prediction results, as shown in Table 8.

We strictly controlled the annotation quality of both data sets. Hence, the probability of causes (1-3) was relatively low. Causes (4-6) were more likely to occur, and cause (7) mainly occurred on some entities that were less common or had fewer training samples.

**Table 8:** Different types of errors on 2 data sets.

Types of errors Illustrations			Example
**(1) Annotation error**			
	1. Some manually annotated entities contained punctuation marks unrelated to the entities.	For instance, some Laboratory entities, like “PLT^a^,” “NEUT^b^,” and “CAE^c^,” on the Yidu-S4K data set contained commas, which were correctly recognized as “PLT,” “NEUT,” and “CAE” in our model.	
	2. A few entity categories were confused.	For example, “PET-CT^d^” was manually annotated as a Laboratory entity on the Yidu-S4K data set, but our model correctly predicted as an Image entity.	
**(2) Inconsistent annotation**			
	The inconsistent annotation will affect the accuracy of machine learning.	On the Yidu-S4K data set, the character “下 (below)” expressing orientation of “剑突下 (below xiphoid)” was not annotated, and the character “部 (part)” expressing the part of “咽喉部 (hypopharynx)” was also not annotated. Most of the characters expressing specific locations were annotated.	
**(3) Missing annotation**			
	The missing annotated entity will also affect the overall effect of the model.	The Disease entity “窦性心律 (sinus rhythm)” was missed annotated on the Yidu-S4K data set, and the Medicine entity “生理盐水 (normal saline)” was missed annotated on the self-annotated data set.	
**(4) Entity with a non-Chinese character symbol**			
	Figures, letters, and other symbols cannot be extracted with more semantic features than Chinese characters. Hence, it may be difficult to recognize entities with symbols other than Chinese characters in the Chinese corpus.	The failure to recognize the non-Chinese character entities, like the Laboratory entity “AFP^e^” on the Yidu-S4K data set and the Examination entity “HCG^f^” on the self-annotated data set, so did such situations as the Medicine entity “VP^g^-16” was recognized as “VP-,” and “50%葡萄糖 (50% glucose)” as “葡萄糖 (glucose)” on the Yidu-S4K data set.	
**(5) Presence of nested entities**			
	On the Yidu-S4K data set, the Disease entity and Image entity might contain the Anatomy entity.	For example, the Disease entity “二尖瓣后叶钙化 (posterior mitral valve leaflet calcification)” was recognized as the Anatomy entity “二尖瓣 (bicuspid),” and the Image entity “腹部彩超 (abdominal color doppler ultrasound)” was recognized as the Anatomy entity “腹部 (abdominal).”	
	On the self-annotated data set, entity nesting is more severe, the Disease entity, Examination entity, and Instrument entity might contain the Anatomy entity, and the Instrument entity might contain the Operation entity.	For example, the Disease entity “内踝骨折 (ankle fracture)” was recognized as the Anatomy entity “内踝 (medial malleolus),” the Examination entity “骨髓组织病理 (bone marrow histopathology)” was recognized as the Anatomy entity “骨髓 (bone marrow),” the Instrument entity “胸部支具 (chest brace)” was recognized as the Anatomy entity “胸(chest),” and the Instrument entity “左胸引流管 (left thoracic drainage tube)” was recognized as the Operation entity “左胸引流 (left thoracic drainage).”	
**(6) More entities with mixed representation**			
	Entity composition is more complex, mixed representations occur more often.	The Medicine entity “奥沙利铂 (乐沙定) (Oxaliplatin [Eloxatin])” on the Yidu-S4K data set was recognized as “奥沙利铂 (Oxaliplatin)” and “乐沙定 (Eloxatin),” respectively, the Disease entity “CD5^h^+弥漫大B细胞淋巴瘤 (白血病期)” on the self-annotated data set was recognized as “CD” and “弥漫大B细胞淋巴瘤 (白血病期) (diffuse large B-cell lymphoma [Leukemia stage]),” and the Examination entity “肥达、外斐反应 (Widal, well-felix reaction)” on the self-annotated data set was recognized as “肥达 (Widal)” and “外斐反应 (well-felix reaction),” respectively.	
**(7) Insufficient entity training data**			
	In the case of insufficient training samples, the machine may provide inadequate training for entities, so that the machine cannot fully learn the features of such entities, failing to recognize many entities.	On the self-annotated data set, the number of Instrument entities was less than that of other categories (Table 2), accounting for only 1.52% of the total, those entities might never appear in the training data set, such as “针筒 (syringe),” “微导管 (microtubule),” “550px碳钢钻头 (550px carbon steel drill bit),” etc.	

^a^PLT: platelet count.

^b^NEUT: neutrophil count.

^c^CAE: carcinoembryonic antigen.

^d^PET-CT: positron emission tomography-computed tomography.

^e^AFP: alpha fetoprotein.

^f^HCG: human chorionic gonadotropin.

^g^VP: etoposide.

^h^CD5: a differentiation antigen, cluster of differentiation 5.

## Discussion

### Principal Findings

In this study, we developed a Chinese CNER method based on multisemantic features. The method extracted the semantic features of text using the RoBERTa-wwm model after dynamic fusion, extracted the fine-grained semantic features of Chinese characters by 1D CNN, and converted Chinese characters into square images to extract the image features of the simplified Chinese characters from another modality by 2D CNN. We conducted a series of experiments to evaluate the model’s performance on the Yidu-S4K data set and self-annotated data set; the results showed that the *F*_1_-scores of the proposed model in this study were 89.28% and 84.61% on the 2 data sets, respectively. The model showed a higher and more stable performance in all experiments and could help recognize entities in most categories. Furthermore, its migrative property and adaptability to different data were acceptable. We also demonstrated that multisemantic features were effective through 2 ablation experiences and analyzed the error cases of NER, which might provide a basis for subsequent studies and standardization of the corpus.

Compared with ensemble models, for the BiLSTM-CRF model, the representation information of characters was obtained with the help of a vector look-up table. However, the information obtained by this method was too simple to excavate the text’s semantic meaning or solve problems such as the polysemy of words. Hence, the model did not perform well. Kong et al [69] constructed a multilayer CNN to obtain short-term and long-term contextual information, and the attention mechanism was used to calculate the weight distribution in each hidden layer so that the features of each coding layer could be fully extracted and used to improve the entity recognition performance. However, this model required numerous radical and dictionary features to complete the semantic supplement of the context. Li et al [70] proposed an ELMo-Lattice-LSTM-CRF model. The ELMo word dynamic representation model could learn complicated word features and the context-based changes of these features, while the lattice structure provided extra entity boundaries and other semantic information for CNER of EMRs through the Word2Vec model and dictionaries. Li et al [41] proposed an ELMo-BiLSTM-CRF model that improved the semantic recognition ability of the machine for text. It reduced problems, such as word polysemy, when compared with the BiLSTM-CRF model and reduced the computational complexity of the lattice structure compared with the ELMo-Lattice-LSTM-CRF model. Moreover, this model could fully use contextual information by replacing LSTM with BiLSTM. Wan et al [71] fine-tuned the ELMo model based on EMRs to achieve embedding for domain-specific text and then used a transformer as an encoder to alleviate the long context–dependent problems and finally achieved CNER through CRF decoding. Wang et al [72] proposed a model for NER based on the LSTM-CRF model by storing and merging characters, words, and other features. However, as the text embedding process of this method is more complicated, it is necessary to create dictionaries of characters and words to obtain multigranularity text features at first and then store and merge the obtained features using a tree structure to achieve text embedding. These methods have achieved a few good results, but our proposed method is still competitive and has the best performance among all the models, as shown in Table 4.

Compared with PLMs related to BERT, both the BERT-BiLSTM-CRF and BiLSTM-CRF models could obtain word-level vector representations. However, the word-level vector obtained by BERT contained rich contextual characteristics, including morphology, syntax, semantics, location, and other important semantic information, which can directly improve the task performance by replacing the lattice structure and complicated text representation methods in Table 4, such as dictionaries of characters and words. Compared with BERT, RoBERTa-wwm used more data for pretraining, and the dynamic wwm allows itself to flexibly learn word-level representation information, which compensates for the shortcomings that BERT can only obtain character-level representation. Thus, richer word-based text representation information could be obtained. Combined with the experimental results in Table 4, the RoBERTa-wwm-BiLSTM-CRF model, without introducing features, outperformed the other ensemble models. Therefore, using the PLM RoBERTa-wwm with a whole word mask can effectively improve the Chinese CNER performance, thus avoiding the use of complex text embedding and feature embedding methods.

In addition, 2 ablation experiments showed that different features and means lead to different degrees of improvement in the semantic comprehension ability of the model. Multisemantic features could help the machine to obtain richer semantic information, whereas dynamic fusion could fully recognize and used the representation information so that the model performance could be comprehensively improved. Considering the heterogeneity among data, using 1 method alone or both methods may affect the generalization ability of the model. In this study, the model combining the fine-grained semantic features and image features of Chinese characters and dynamic fusion might not show the best performance. However, it was more universal and could maintain the performance at a relatively high level compared with other experimental models. Furthermore, introducing more feature engineering was conducive to fully mining the semantic information of text connotation with the help of fine-grained semantic information contained in Chinese characters and improving the performance of the model on different data sets through the cross-complementarity of different features in a relatively stable manner.

To reduce the error rate of entity recognition, specifically for human-caused errors, we could try to avoid these problems by further improving the annotation quality. For the data special characteristics or data defects, the errors might be reduced by medical knowledge, medical dictionaries, and some rules, regardless of the lack of training data.

### Limitations and Future Work

The limitation of this study was that the ratio of the 6 entity types on the Yidu-S4K data set did not exactly follow 7:3, such that the ratio of the training set to test set for disease entities is approximately 0.7610:0.2390; the ratio of the training set to test set for medicine entities is approximately 0.7898:0.2102; and the ratio of the training set to test set for all entities is approximately 0.7463:0.2537. The unbalanced data of different entity types in the training and test sets caused a performance bias. Although the ratio of the training set to the test set of the EMRs was 7:3, we could not ensure that the number of entities of each type in each EMR in the training set and test set remained at a similar ratio.

In the future, we may focus on the recognition of a specific entity type in EMRs to improve the CNER performance. In addition, we will incorporate other prior medical knowledge or assign different weights to the Chinese character semantic features and image features, such as using the attention mechanism to capture important features, to improve the performance of the model.

### Conclusions

This study proposes a Chinese CNER method to learn a semantics-enriched representation of Chinese character features in EMRs to enhance the specificity and diversity of feature representations. The results showed that the model had state-of-the-art performance on 2 Chinese CNER data sets compared with existing models. We demonstrated that multisemantic features could provide richer and more fine-grained semantic information for Chinese CNER through the cross-complementarity of different semantic features. This enabled the model to learn a better feature representation and improve its generalization ability.

## Abbreviations

BERT: Bidirectional Encoder Representation from Transformers
BiLSTM: Bidirectional Long Short-Term Memory
CNER: clinical named entity recognition
CNN: convolutional neural network
CRF: conditional random fields
ELMo: Embeddings from Language Models
EMR: electronic medical record
FN: false negative
FP: false positive
LSTM: long short-term memory
NER: named entity recognition
PLM: pretrained language model
RoBERTa: Robustly Optimized Bidirectional Encoder Representation from Transformers Pretraining Approach
TP: true positive
Word2Vec: Word to Vector
wwm: Whole Word Masking
